# Miniature Erupting Volcano-Shaped Mitral Valve Aneurysm Secondary to *Streptococcus agalactiae* ST1656 Endocarditis: A Case Report

**DOI:** 10.3389/fcvm.2021.728792

**Published:** 2021-08-19

**Authors:** Hiroyuki Yamamoto, Hiroyuki Yamada, Takahiro Maeda, Mieko Goto, Yoshihiko Ikeda, Takashi Takahashi

**Affiliations:** ^1^Department of Cardiovascular Medicine, Narita-Tomisato Tokushukai Hospital, Chiba, Japan; ^2^Department of Cardiovascular Surgery, Narita-Tomisato Tokushukai Hospital, Chiba, Japan; ^3^Laboratory of Infectious Diseases, Graduate School of Infection Control Sciences and Omura Satoshi Memorial Institute, Kitasato University, Tokyo, Japan; ^4^Department of Pathology, National Cerebral and Cardiovascular Center, Suita, Japan

**Keywords:** GBS IE, MVA, MR, diabetes, TEE, ST1656

## Abstract

Mitral valve aneurysm (MVA) is a rare but life-threatening valvular pathologic entity most commonly associated with infective endocarditis (IE) of the aortic valve (AV). We describe a diabetic patient with ruptured anterior MVA secondary to capsular genotype V *Streptococcus agalactiae* (GBS) harboring novel ST1656 IE without AV involvement. Our patient presented with manifestations of various serious systemic and intracardiac complications, requiring early surgery, but ultimately died from non-cardiogenic causes. This case emphasizes the importance of treating MVA as a dangerous sequela of IE, of performing transesophageal echocardiography to make its accurate diagnosis and institute early surgical intervention, and of considering GBS as a rare but important causative agent of IE in elderly patients with comorbidities.

## Introduction

Mitral valve aneurysm (MVA), a saccular outpouching of the mitral leaflet toward the left atrium (LA), is rare but can be a fatal sequela of active or previous aortic valve (AV) infective endocarditis (IE), causing aneurysmal rupture and systemic embolization (1–3). However, accurate diagnosis in the absence of these clinical features remains challenging.

## Case Description

**Figure 1:**
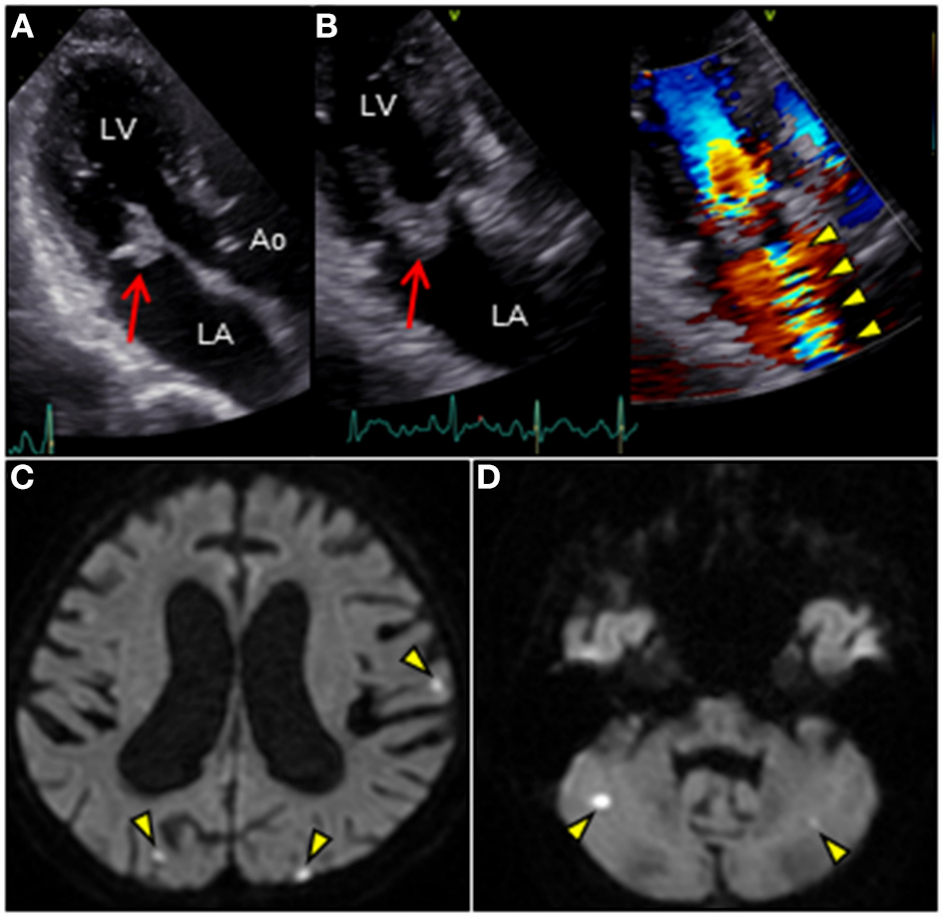
**(A,B)** Transthoracic echocardiography in apical three-chamber view reveals an irregular mass (18 × 17 mm, arrow) attached to the atrial side of the anterior mitral leaflet. Color Doppler image reveals massive mitral regurgitation traversing the mass (arowheads). **(C,D)** Brain diffusion-weighted magnetic resonance imaging reveals multiple acute hyperintense ischemic strokes in both hemispheres (arrowheads). Ao, aorta; LA, left atrium; LV, left ventricle.

**Figure 2:**
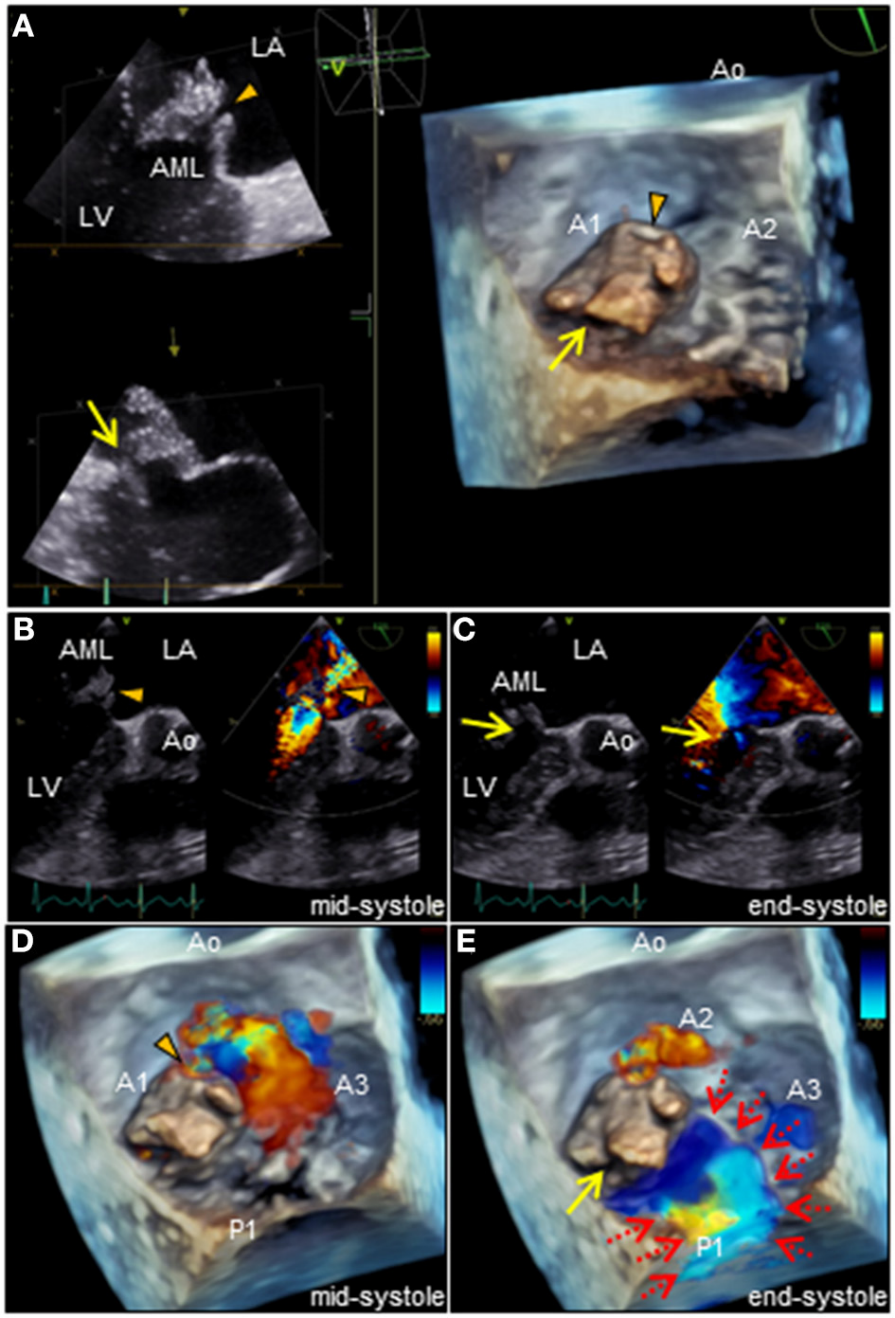
**(A)** Three-dimensional TEE of the mitral valve. Note the two distinct ruptured perforations through the MVA (arrowhead and arrow, respectively). **(B–E)** Two or three-dimensional color Doppler TEE reveals that severe MR with two different jets communicate with the LA through the MVA: a superior jet (arrowhead) and a posterior jet (arrow), respectively. Note the MR with posterior jet heading toward the LA via the PML surface (dotted arrows). AML, anterior mitral leaflet; Ao, aorta; LA, left atrium; LV, left ventricle; MR, mitral regurgitation; PML, posterior mitral leaflet; MVA, mitral valve aneurysm, TEE, transesophageal echocardiography.

**Figure 3:**
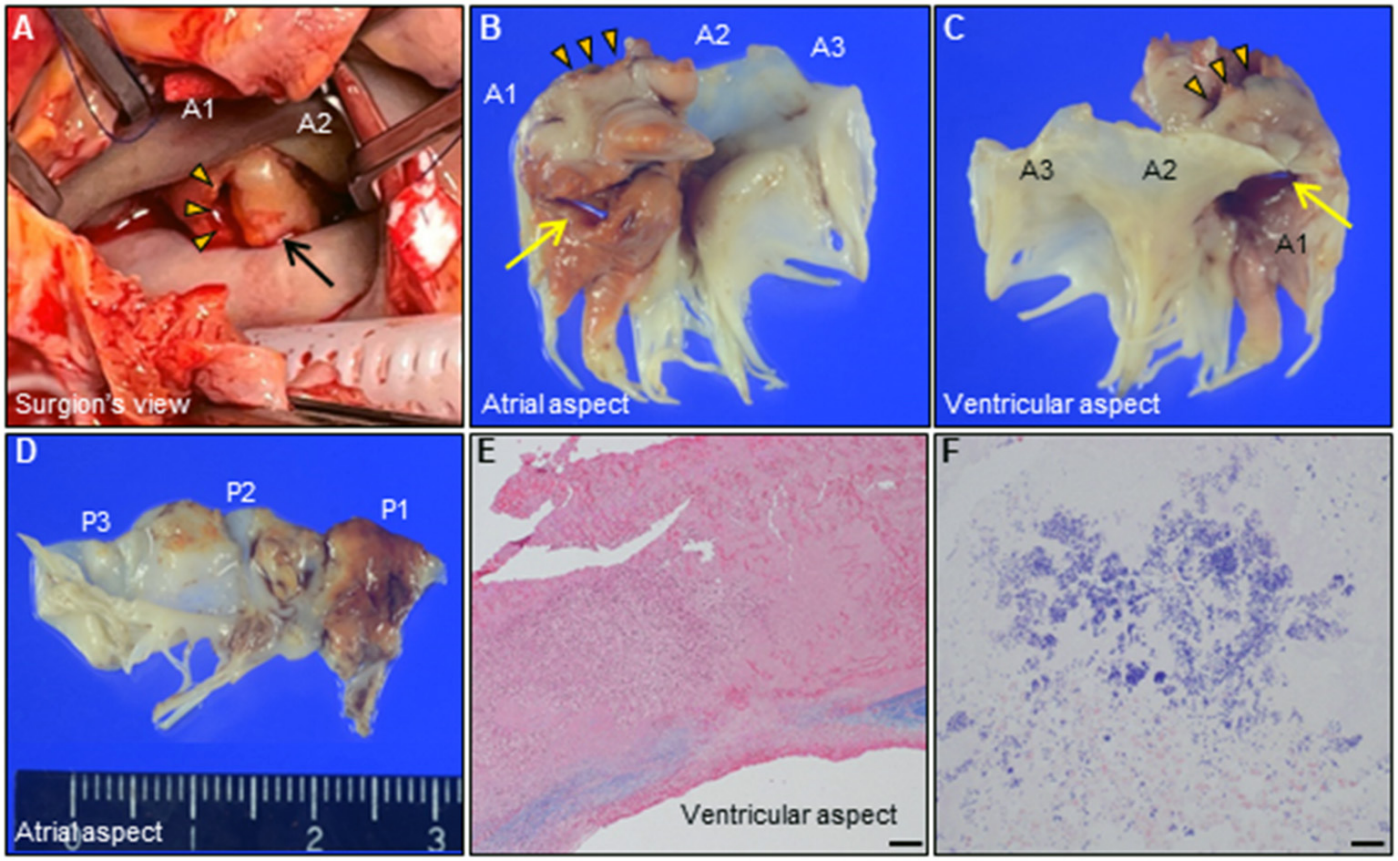
**(A)** Intraoperative photograph of the mitral valve. Giant MVA (arrow, 28 × 23 × 12 mm) with ruptured perforation (arrowheads) is observed on the AML in A1. **(B–D)** Representative photographs of the extracted mitral valve leaflets [**(B,C)**, AML; **(D)**, PML]. Note the two distinct perforations through the MVA (arrow, arrowheads, respectively). **(E,F)** Histological findings of the resected MVA shows extensive valvular destruction with a high degree of neutrophilic inflammatory cell infiltration and bacterial agglomeration. [**(E)**, Elastica van Gieson staining, Bar 200 μm; **(F)**, Gram staining, 20 μm]. AML, anterior mitral leaflet; MVA, mitral valve aneurysm; PML, posterior mitral leaflet.

A 69-year-old man was admitted to our hospital for lethargy. He reported a 1-month history of repetitive watery diarrhea before admission. He had no history of prior cardiac disease but did have a history of hypertension and alcoholism. On admission, physical examination results were as follows: blood pressure, 76/51 mmHg; mild hypothermia: 36.0°C; tachycardia, 115 beats/min; and grade 4/6 holosystolic murmur at the cardiac apex. He was drowsy, but neurologic examination was unremarkable. A collapsed jugular vein and cool skin were noted. The electrocardiogram revealed sinus tachycardia. The chest radiograph was unremarkable. Laboratory testing revealed elevated inflammatory markers; severe thrombocytopenia; acute renal failure (RF); and elevated levels of liver enzymes, blood glucose, hemoglobin A1c, and brain natriuretic peptide, suggesting systemic inflammation. Transthoracic echocardiography (TTE)

revealed an irregular mobile mass with a wide base attached to the atrial side of the anterior mitral leaflet (AML) (Figure 1A). Color Doppler imaging revealed severe mitral regurgitation (MR) with high-velocity turbulent jet across the mass, leading to a suspicion of vegetations (Figure 1B; Supplementary Video 1). Differential diagnoses included chordal rupture, cardiac tumors including myxoma, cysts of the mitral valve (MV), and vegetations. Brain magnetic resonance imaging revealed acute multiple cerebral infarctions, suggesting systemic embolism (Figures 1C,D). Thus, a presumptive diagnosis of MV IE complicated by cerebral embolization was made. The patient was in a state of shock and was transferred to the intensive care unit for careful monitoring and management, including hydration, platelet transfusion, strict glycemic control, and intravenous empiric antimicrobial therapy (vancomycin, 500 mg/day IV; meropenem, 2 g/day IV). On day 4, the patient's clinical condition deteriorated abruptly due to acute heart failure (HF), requiring intravenous furosemide (20 mg twice daily), infusions of dobutamine (2 μg/kg/min) and human atrial natriuretic peptide (0.0125 μg/kg/min) to ameliorate pulmonary congestion. Subsequent transesophageal echocardiography (TEE) revealed a saccular bulge of the thickened AML in A1, characterized by protrusion toward the LA with systolic expansion, and diastolic collapse into the left ventricle, consistent with MVA (Figure 2A; Supplementary Video 2). Color Doppler imaging further clarified morphological details of the MVA and the spatial orientation of the MR jet (Figures 2B–E; Supplementary Videos 3, 4). Severe MR with two distinct jets communicating with the LA through the MVA were noted: a superior jet from the center of the MVA, and a posterior jet from the side of the MVA. The appearance was reminiscent of a volcano erupting. Notably, MR with posterior jet reached the posterior mitral leaflet (PML), raising major concerns of mitral annular abscesses, but there were no typical echocardiographic findings of abscess. Furthermore, there was neither regurgitation nor vegetations of the AV. The blood and diarrheal stool cultures performed on admission showed the growth of two *Streptococcus agalactiae* strains (Lancefield carbohydrate group B *Streptococcus*, GBS), meeting a modified Duke criterion for a diagnosis of IE. On day 5, semi-urgent cardiac surgery was performed following a multidisciplinary team discussion. Surgical findings included a large mass of vegetations on the A1 scallop of the MV with extensive valve destruction, which formed an aneurysm through which two ruptured perforations had occurred (Figures 3A–C). We confirmed the spread of infection onto the atrialis of the PML without evidence of perivalvular abscess (Figure 3D). Nevertheless, all of the AV leaflets and the sinus of Valsalva were intact. Subsequently, the patient underwent MV replacement with a prosthetic valve (Epic 27 mm, St. Jude Medical). Finally, the GBS infection was confirmed through pathologic examination and polymerase chain reaction (Figures 3E,F; Supplementary Figure 1). Microbiologic analysis (including genotypic features) demonstrated bacterial genomic DNAs extracted from the blood-origin strain, and excised PML tissue showed a perfect match to the sequences of GBS, which belonged to capsular genotype V and a novel sequence type (ST) assigned to ST1656 (Supplementary Text 1). Based on antimicrobial susceptibility testing results with the blood-origin strain, antimicrobial de-escalation was performed with high-dose penicillin G (4 million units IV every 4 h). Postoperatively, the patient was stable without HF exacerbation. However, on day 20, the patient developed active multiple gastric ulcers, followed by acute cholangitis and acute RF. Despite intensive treatment, he died on day 40.

**Figure 4:**
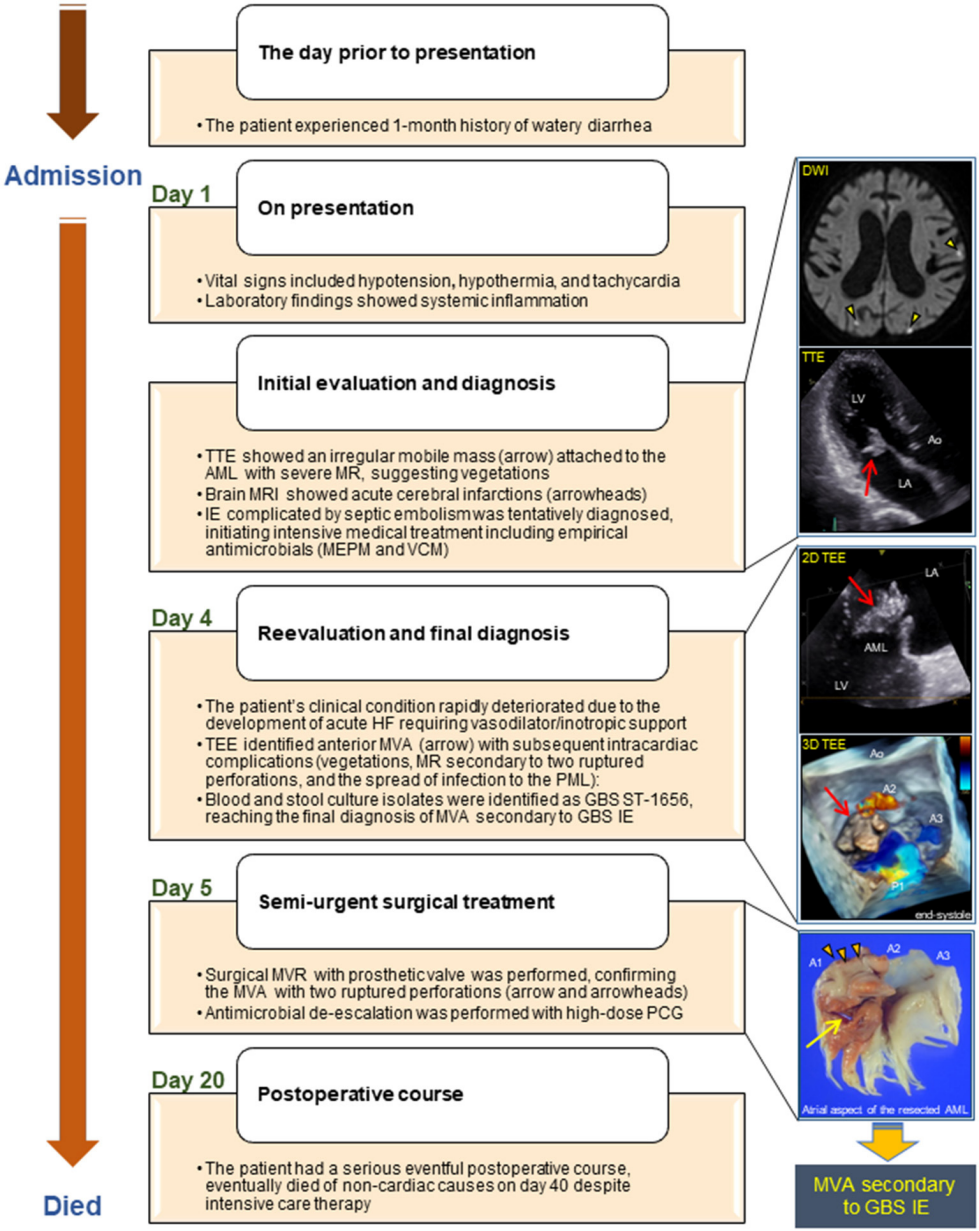
Illustrated timeline of the case. AML, anterior mitral leaflet; Ao, aorta; DWI, diffusion-weighted image; GBS, *Streptococcus agalactiae*; HF, heart failure; IE, infective endocarditis; LA, left atrium; LV, left ventricle; MRI, magnetic resonance imaging; MEPM, meropenem; MR, mitral regurgitation; MVA, mitral valve aneurysm; MVR, mitral valve replacement; PCG, penicillin G; PML, posterior mitral leaflet; ST, sequence type; TEE, transesophageal echocardiography; TTE, transthoracic echocardiography; VCM, vancomycin.

A summarized illustration of the case presentation is provided in Figure 4.

## Discussion

To our knowledge, we describe here, the first case of native MV IE caused by a GBS genotype V/ST1656 strain without AV involvement. Subsequently, the patient developed MVA secondary to GBS IE concurrent with various fatal intracardiac and systemic complications, eventually died despite aggressive antimicrobial and surgical management. Our case may provide four important clinical suggestions.

First, we describe a case of MVA secondary to IE without AV involvement.

MVA is a rarely encountered echocardiographic finding, identified in <0.3% in the TEE setting (1–3). AV IE is the most common causative factor in MVA since infectious stimuli may allow direct or indirect injury to the leaflet of the MV. Nevertheless, the AV was not involved by IE in our case, which is an uncommon setting. Recently, the frequency of diagnosis of MVA has been increasing because of advances in imaging and increased awareness of this disease. Moreover, MVA of non-infectious etiology has been reported; these causes include connective tissue diseases, Libman-Sacks endocarditis, and degenerative changes (4–6), suggesting that this valvular pathology is no longer rare. Our case highlights the significance of considering MVA as a possible complication of IE, even without AV involvement.

Second, our case of MVA revealed various serious intracardiac complications requiring early cardiac surgery.

MVA can be manifested in various ways, ranging from an asymptomatic presentation to fatality due to complications.

Aneurysmal rupture and systemic embolization are the most common complications of MVA (7). Although it is rare in patients with MV IE, perivalvular abscess is the most feared complication with a high mortality (8). However, because the signs can be non-specific, early diagnosis and timely surgery are often not possible. Our patient presented with ruptured anterior MVA secondary to IE, complicated by multiple embolic strokes, acute HF caused by severe MR, and the spread of infection through the PML, almost resulting in perivalvular abscesses. Subsequently, the patient required semi-urgent surgery. Unfortunately, the initial TTE missed MVA in our case, but MR with high-velocity turbulent jet might have been a red flag that could have raised suspicion of MVA.

As the third clinical suggestion, TEE was found most suitable for the definitive diagnosis of MVA, detection of its complications, and surgical planning in the present case.

TEE is of significant diagnostic value in detecting intracardiac complications such as MVA and leaflet perforation, compared to TTE (sensitivity, 95 vs. 45%; specificity, 92.9 vs. 68%, respectively) (9). The unique saccular bulge of the AML expanding toward the LA with systolic expansion and diastolic collapse into the left ventricle observed on TEE enabled easy differentiation from vegetations or other mass, leading to the correct diagnosis. Also, TEE has excellent capability of detecting perivalvular abscess (87% sensitivity, 94.6% specificity, 90.9% positive predictive value, and 92.1% negative predictive value) (8). The absence of findings of perivalvular abscess confirmed on TEE was helpful in our surgical planning. Moreover, three-dimensional color Doppler TEE enabled us to clearly visualize the spatial relationship between MVA, two ruptured perforations, and MR jets traversing the MVA. This appearance resembled a miniature active volcanic eruption in our case. Especially, the direction of MR jets can be an important indicator of the mechanism and possible complications of MVA. TEE findings suggested that the ruptured anterior MVA secondary to IE resulted in MR with posterior jet, which impacted on the atrialis of the PML and produced a secondary site of infection, findings which were confirmed pathologically. Although, direct extension of vegetations is generally believed to be the main mechanism of perivalvular abscess in patients with MV IE, in our case, MR with posterior jet might have posed a crucial threat of perivalvular abscess.

The fourth clinical suggestion is that this case emphasized the importance of early diagnosis of GBS IE.

Our case with GBS IE revealed serious systemic complications (e.g., HF, septic shock, RF, and cerebral embolism) and intracardiac complications (e.g., MVA, vegetations, secondary MR due to ruptured perforations, and spread of infection to the PML) requiring early surgery. Our case raises three clinical challenges for GBS IE.

The first clinical challenge is the management of GBS IE.

GBS IE is rare, with an incidence of about 2–3% of all cases of IE, and is characterized by community-acquired infections that often involve native valves of patients even in the absence of pre-existing valvular heart disease (10–12). Vegetations are often large and fragile, progressively deteriorating with extensive valve destruction, and associated with a high incidence of systemic embolism (37–51%) and a high mortality rate (29–47%). Thus, the aggressive behavior of GBS IE is comparable to that of *Staphylococcus aureus* IE. Factors associated with mortality in GBS IE include HF [Odds Ratio (OR): 9.500, 95% Confidence Interval (CI): 1.73–3.5, *p* = 0.029], neurological impairment (OR: 8.000, 95% CI: 1.399–45.756, *p* = 0.023), and RF (OR: 5.750, 95% CI: 1.124–29.411, *p* = 0.041) (12). Similar conditions (including HF and RF) might have had an impact on our patient's eventful postoperative course. In view of the reports of improved prognosis with early surgery (13), clinicians should recognize this rare entity as a virulent disease and establish an optimal timing of surgery well in advance of serious complications ensuing.

The second clinical challenge is to consider GBS IE in the differential diagnosis.

In general, GBS, which commonly colonizes human genital and gastrointestinal tracts, is known as the leading cause of neonatal sepsis and meningitis (14). Recently, cases of infection in the elderly or in patients with comorbidities (e.g., alcoholism, diabetes, liver cirrhosis, or malignancy) have been increasingly reported (12, 15, 16). Among them, diabetes is the most common comorbidity in GBS IE (35%). The repeated episodes of diarrhea suggest that the gastrointestinal tract was the route of infection in our case. Our patient exhibited several risk factors for invasive GBS infections including untreated diabetes, as described above. Thus, these circumstances might have had an influence on the onset and severity of GBS IE in our case although the exact mechanism of GBS IE remains unclear. Therefore, a high index of suspicion should be exercised for GBS as a possible causative pathogen of severe IE among elderly patients with comorbidities.

The third clinical issue concerns the importance of the new clone of GBS identified in our case.

We searched for the epidemiological features of GBS IE registered on the Pubmlst database (*n* = 19, including our clones) (Supplementary Tables 1, 2). The prevalent clones of serotype/ST were III/ST19 (*n* = 4) from Japan/Australia, Ib/ST10 (*n* = 4) from Japan, V/ST1 (*n* = 3) from Japan/Australia, and VIII/ST1 (*n* = 2) from Japan. Therefore, the genotype V/ST1656 from both GB125–2 (blood) and GB125–3 (PML) in our case seems to be a very rare clone. The serotype III/ST19 clone is highly virulent and associated with very severe GBS IE even in healthy adult patients (17). Since our patient had several risk factors for invasive GBS infections, the pure virulence potential of our new clone is still unclear; thus, further data accumulation and analysis are necessary.

## Conclusion

We describe a fatal case of MVA secondary to GBS IE, complicated by variable intracardiac and systemic complications, requiring early surgical intervention.

MVA is a rare but potentially fatal sequela of IE. Therefore, it is imperative to detect MVA early and perform surgery expediently. TEE is useful for an accurate diagnosis and optimal surgical planning. Therefore, clinicians should be aware of this rare valvular anomaly and should not hesitate to perform TEE in patients with potential IE. In addition, our case highlights the clinical significance of bearing in mind GBS IE as a rare cause of severe IE.

## Data Availability Statement

The original contributions presented in the study are included in the article/Supplementary Material, further inquiries can be directed to the corresponding author/s.

## Ethics Statement

The authors confirm that written informed consent for submission and publication of this case report, including the images and associated movie, has been obtained from the patient's family.

## Author Contributions

HYamam contributed to the clinical design and concept. HYamam and HYamad acquired the clinical data. TM and MG performed microbiological analyses. YI performed pathological analyses. HYamam and TT interpreted the data and drafted and revised the manuscript. All authors discussed, read, and approved the manuscript and its submission for publication.

## Conflict of Interest

The authors declare that the research was conducted in the absence of any commercial or financial relationships that could be construed as a potential conflict of interest.

## Publisher's Note

All claims expressed in this article are solely those of the authors and do not necessarily represent those of their affiliated organizations, or those of the publisher, the editors and the reviewers. Any product that may be evaluated in this article, or claim that may be made by its manufacturer, is not guaranteed or endorsed by the publisher.

## Abbreviations

MVA: mitral valve aneurysm
LA: left atrium
AV: aortic valve
IE: infective endocarditis
TTE: transthoracic echocardiography
AML: anterior mitral leaflet
MR: mitral regurgitation
MV: mitral valve
HF: heart failure
TEE: transesophageal echocardiography
PML: posterior mitral leaflet
GBS: *Streptococcus agalactiae*
ST: sequence type
OR: odds ratio
CI: confidence interval.
