# Supplementary material for: Miniature Erupting Volcano-Shaped Mitral Valve Aneurysm Secondary to Streptococcus agalactiae ST1656 Endocarditis: A Case Report
Source: Front Cardiovasc Med. 2021 Aug 19;8:728792. doi: 10.3389/fcvm.2021.728792 (PMC8416758; doi:10.3389/fcvm.2021.728792)
Supplement: Supplementary file 5 [file Data_Sheet_1.pdf]

Supplementary Material

TEXT LEGENDS

Text 1. Microbiological analyses

The stored strains from diarrheal stool (GB125–1) and blood (GB125–2) samples in addition to the tissue fragment of the posterior mitral leaflet (PML) having vegetations (GB125–3, **Supplementary Figure 1A**) were immediately sent to the Laboratory of Infectious Diseases, Ōmura Satoshi Memorial Institute, Kitasato University.

A tissue specimen excised from the PML was subjected to Gram staining examination (1) and isolation culture. Microscopic findings of the stained images demonstrated Gram-positive round-shaped microorganisms clustered in chains (**Supplementary Figure 1B**). However, because no bacteria were isolated using the tissue culture, we obtained a DNA sample (GB125–3) extracted using a DNeasy Blood & Tissue Kit (Qiagen, Germany) after pretreating it with both lysozyme (Thermo Fisher Scientific, USA) and proteinase K (Qiagen) (2). We measured the DNA concentration as 21.6 ng/mL using a Nanodrop Lite spectrophotometer (Thermo Fisher Scientific). **Supplementary Table 1** shows the phenotypic and genotypic characteristics of two strains (GB125–1 and GB125–2) and a DNA sample extracted from the PML (GB125–3). Type strain American Type Culture Collection 13813 of *S. agalactiae* (isolated from environmental milk) was applied as a quality control for the phenotypic and genotypic analyses (3).

The phenotypic assays included assessment of gross appearance of colonies on a sheep blood agar plate, carbohydrate group (Lancefield cell wall antigenicity), biofilm formation ability using crystal violet staining (absorbance at 545 nm, mean $\pm$ standard deviation of 5 wells), and antimicrobial resistance phenotype. The genotypic assays included percent similarity to

the type strain using 16S rRNA sequencing, amplification of *S. agalactiae*-specific gene *dltS* encoding histidine kinase (sensor protein in the membrane), capsular genotype, sequence type (ST) (allelic profile, *adhP–pheS–atr–glnA–sdhA–glcK–tkt*), virulence-associated gene profile (*bca–rib–bac–lmb–cylE–hylB–pavA–pilB–spb1–srtC1–brpA*), and genotype contributing to resistance to macrolide/lincosamide class [*erm*(A)–*erm*(B)–*mef*(A)–*erm*(F)] and tetracycline class [*tet*(M)–*tet*(O)–*tet*(K)–*tet*(L)–*tet*(S)–*tet*(Q)], based on polymerase chain reaction (1,3). The features of phenotypes (biofilm formation ability and others) and genotypes (capsular genotype V, ST1524, and virulence-associated gene profile of *bca–rib–lmb–cylE–hylB–pavA–brpA* without antimicrobial resistance genotype) of GB125–1 were found to be similar to those of GB125–2 (including the genotypic features of GB125–3). A novel ST1656 (92–1–4–4–1–3–2) is a single locus variant of ST1524 (1–1–4–4–1–3–2) forming the clonal complex.

References

1. Shibayama A, Yoshizaki T, Tamaki M, Goto M, Takahashi T. Pyogenic sternoclavicular arthritis caused by *Streptococcus agalactiae* in an elderly adult with diabetes mellitus. J Am Geriatr Soc (2016) 64:1376-7. doi: 10.1111/jgs.14169.

2. Fukushima Y, Murata Y, Katayama Y, Tsuyuki Y, Yoshida H, Mizutani T, et al. Draft genome sequence of blood-origin *Streptococcus canis* strain FU149, isolated from a dog with necrotizing soft tissue infection. Microbiol Resour Announc (2020) 9: e00737-20. doi: 10.1128/MRA.00737-20.

3. Maeda T, Takayama Y, Fujita T, Taniyama D, Tsuyuki Y, Shibayama A, et al. Comparison between invasive and non-invasive *Streptococcus agalactiae* isolates from human adults, based on virulence gene profile, capsular genotype, sequence type, and antimicrobial resistance pattern. Jpn J Infect Dis doi: 10.7883/yoken.JJID.2020.761. (in press)

SUPPLEMENTARY FIGURE LEGENDS

Figure 1. The surgically removed posterior mitral valve leaflet and pathology

(A) Tissue fragment of the posterior mitral leaflet and (B) Gram staining images of the homogenized posterior mitral leaflet. Microscopic findings of the staining images demonstrated Gram-positive round-shaped microorganisms clustered in chains. Bars 1 mm and 5 μm, respectively.

SUPPLEMENTARY TABLE LEGENDS

Epidemiological features of clones causing GBS (*Streptococcus agalactiae*) endocarditis registered on the PubMLST database (https://pubmlst.org/bigsdb?db=pubmlst_sagalactiae_isolates&page=query, isolate number 11,859 as of 25–Mar–2021)

Table 1. Phenotypic and genotypic features of GBS isolated from the present case

Table 1

Sample	GB125-1	GB125-2	GB125-3[b]
Collection date (year/month/day)	2021/Feb/19	2021/Feb/19	2021/Feb/25
Clinical specimen	Diarrheal stool	Blood	Posterior mitral leaflet
Gross appearance of colonies on a sheep blood agar plate	Non-mucoid, β-hemolytic small gray-white-colored smooth colonies	Non-mucoid, β-hemolytic small gray-white-colored smooth colonies	NA because of no bacterial isolation using tissue culture
Carbohydrate group (Lancefield cell wall antigenicity)	Group B	Group B	NA
Similarity (%) to the *S. agalactiae* type strain using 16S rRNA sequencing (sequencing size, bp)	100 (599)	100 (595)	100 (599)
S. agalactiae-specific gene, *dltS* encoding histidine kinase (sensor protein in the membrane)	Amplified	Amplified	Amplified
Capsular genotype	V	V	V
ST (allelic profile, *adhP-pheS-atr-glnA-sdhA-glcK-tkt*)	ST1524 (1-1-4-4-1-3-2)	New ST1656 (92-1-4-4-1-3-2)	New ST1656 (92-1-4-4-1-3-2)
Virulence-associated gene profile (*bca-rib-bac-lmb-cylE-hylB-pavA-pilB-spb1-srtC1-brpA*)	*bca-rib-lmb-cylE-hylB-pavA-brpA*	*bca-rib-lmb-cylE-hylB-pavA-brpA*	*bca-rib-lmb-cylE-hylB-pavA-brpA*
Biofilm formation ability assessed using crystal violet staining (absorbance at 545 nm, mean ± SD of 5 wells)[a]	0.04 ± 0.015	0.06 ± 0.015	NA

Antimicrobial resistance phenotype	NA	None	NA
Gene contributing to resistance to macrolide/lincosamide/tetracycline classes	None	None	None

Abbreviations

NA, Not available; SD, Standard deviation; ST, Sequence type.

Genes bca, bac, rib, lmb, cylE, hylB, pavA, pilB, spb1, srtC1, and brpA encode the α and β C protein, Rib protein, laminin-binding protein, one of cylX-K, hyaluronate lyase, fibronectin-binding protein, backbone proteins of pilus island (PI)-2a/PI-2b, pilus-associated sortase C1 of PI-1, and biofilm regulatory protein A, respectively. [a]Type strain American Type Culture Collection 13813 of *S. agalactiae* was applied as a control for phenotypic and genotypic analyses. [b]GB125-3 (the tissue fragment of posterior mitral leaflet) was DNA sample extracted using a DNeasy Blood & Tissue Kit (Qiagen, Germany) after pretreating it with both lysozyme (Thermo Fisher Scientific, USA) and proteinase K (Qiagen). We measured a DNA concentration of 21.6 ng/μL using a Nanodrop Lite spectrophotometer (Thermo Fisher Scientific).

Table 2. Epidemiological features of GBS causing endocarditis in the Isolate Database on the MultiLocus Sequence Typing website

Table 2

Year	Country (region)	Sex/age year	Disease(s)	Source	Epidemiology	Antimicrobial susceptibility data	Capsular serotype/genotype	ST	Allelic profile, *adhP-pheS-atr-glnA-sdhA-glcK-tkt*
2006	Japan (Nagano)	Male/39	Endocarditis	Blood	Community-acquired	Penicillin MIC 0.031	III	ST19	1-1-3-2-2-2-2
2006	Japan (Kanagawa)	Female/52	Endocarditis	Blood	Community-acquired	Penicillin MIC 0.063	III	ST19	1-1-3-2-2-2-2
2006	Japan (Aichi)	Female/65	Endocarditis	Blood	Community-acquired	Penicillin MIC 0.063	Ib	ST10	9-1-4-1-3-3-2
2006	Japan (Iwate)	Female/27	Endocarditis	Blood	Community-acquired	Penicillin MIC 0.063	VIII	ST1	1-1-2-1-1-2-2
2008	USA (MD)	NA/28	Endocarditis, meningitis	Blood	NA	Erythromycin MIC >32, tetracycline MIC >16, clindamycin MIC >32, penicillin MIC 0.06	Ib	ST598	4-3-4-1-3-3-2
2009	France (Ile de France)	Female/66	Endocarditis	Blood	NA	Erythromycin disc testing resistant, tetracycline disc testing resistant, clindamycin disc testing resistant,	V	ST567	106-1-3-1-1-2-2

						penicillin disc testing susceptible			
2010	France (Rhone Alpes)	Female/77	Endocarditis	Blood	NA	Erythromycin disc testing susceptible, tetracycline disc testing resistant, clindamycin disc testing susceptible, penicillin disc testing susceptible	II	ST569	9-1-1-1-3-1-2
2010	Japan (Fukuoka)	Male/30	Endocarditis	Blood	Community-acquired	Clindamycin MIC 0.25, penicillin MIC 0.063, levofloxacin MIC 64	Ib	ST10	9-1-4-1-3-3-2
2010	Japan (Hiroshima)	Female/61	Endocarditis	Blood	Community-acquired	Clindamycin MIC 0.125, penicillin MIC 0.063	NA	ST23	5-4-6-3-2-1-3
2010	Japan (Hiroshima)	Female/66	Endocarditis	Blood	Community-acquired	Clindamycin MIC 0.125, penicillin MIC 0.063	VIII	ST1	1-1-2-1-1-2-2
2010	Japan (Fukuoka)	Male/75	Endocarditis, meningitis, cellulitis	Blood	Community-acquired	Clindamycin MIC 0.25, penicillin MIC 0.063, levofloxacin	Ib	ST10	9-1-4-1-3-3-2

						MIC 64			
2010	Japan (Gifu)	Male/62	Endocarditis	Blood	Community-acquired	Clindamycin MIC 16, penicillin MIC 0.031	III	ST19	1-1-3-2-2-2-2
2010	Japan (Shizuoka)	Male/73	Endocarditis	Blood	Community-acquired	Clindamycin MIC 0.031, penicillin MIC 0.031	V	ST1	1-1-2-1-1-2-2
2010	Japan (Hokkaido)	Male/68	Endocarditis	Blood	Community-acquired	Clindamycin MIC 0.25, penicillin MIC 0.063, levofloxacin MIC 64	Ib	ST10	9-1-4-1-3-3-2
2010	Japan (Ibaraki)	Male/63	Endocarditis	Blood	Community-acquired	Clindamycin MIC 0.5, penicillin MIC 0.063	V	ST1	1-1-2-1-1-2-2
2016	Australia (NSW)	Male/63	Bacteraemia, endocarditis	Blood	Community-acquired	Erythromycin disc testing resistant, tetracycline disc testing resistant, clindamycin disc testing resistant, penicillin disc testing susceptible	V	ST1	1-1-2-1-1-2-2

2018	Australia (NSW)	Female/88	Bacteraemia, endocarditis	Blood	Community-acquired	Erythromycin disc testing susceptible, tetracycline disc testing resistant, clindamycin disc testing susceptible, penicillin disc testing susceptible	III	ST19	1-1-3-2-2-2-2
2021 (GB125-2)	Japan (Chiba)	Male/69	Endocarditis	Blood	Community-acquired	Erythromycin disc testing susceptible, tetracycline disc testing susceptible, clindamycin disc testing susceptible, penicillin disc testing susceptible	V	ST1656	92-1-4-4-1-3-2
2021 (GB125-3)	Japan (Chiba)	Same above	Endocarditis	Posterior mitral leaflet	Community-acquired	NA	V	ST1656	92-1-4-4-1-3-2

Abbreviations

MD, State of Maryland; MIC, Minimum inhibitory concentration; NSW, New South Wales; NA, Not available; ST, Sequence type. Gray shading indicates the epidemiological features in the present case presentation.

SUPPLEMENTARY VIDEO LEGENDS

Video 1. Color Doppler transthoracic echocardiography in apical three-chamber view

Note the severe mitral regurgitation with high-velocity turbulent jet across the mass.

Video 2. Three-dimensional transesophageal echocardiography

A unique aneurysm of the thickened anterior mitral leaflet corresponding to expansion toward the left atrium at systole and collapse at diastole into the left ventricle.

Video 3. Two-dimensional color Doppler transesophageal echocardiography

Color Doppler transesophageal echocardiography in the mid-esophageal three-chamber view shows the two distinct mitral regurgitant jets through the mitral valve aneurysm.

Video 4. Three-dimensional color Doppler transesophageal echocardiography

Note the direction of the two different mitral regurgitant jets communicating with the left atrium through the mitral valve aneurysm.
