# Supplementary figures and images for: Miniature Erupting Volcano-Shaped Mitral Valve Aneurysm Secondary to Streptococcus agalactiae ST1656 Endocarditis: A Case Report
Source: Front Cardiovasc Med. 2021 Aug 19;8:728792. doi: 10.3389/fcvm.2021.728792 (PMC8416758; doi:10.3389/fcvm.2021.728792)

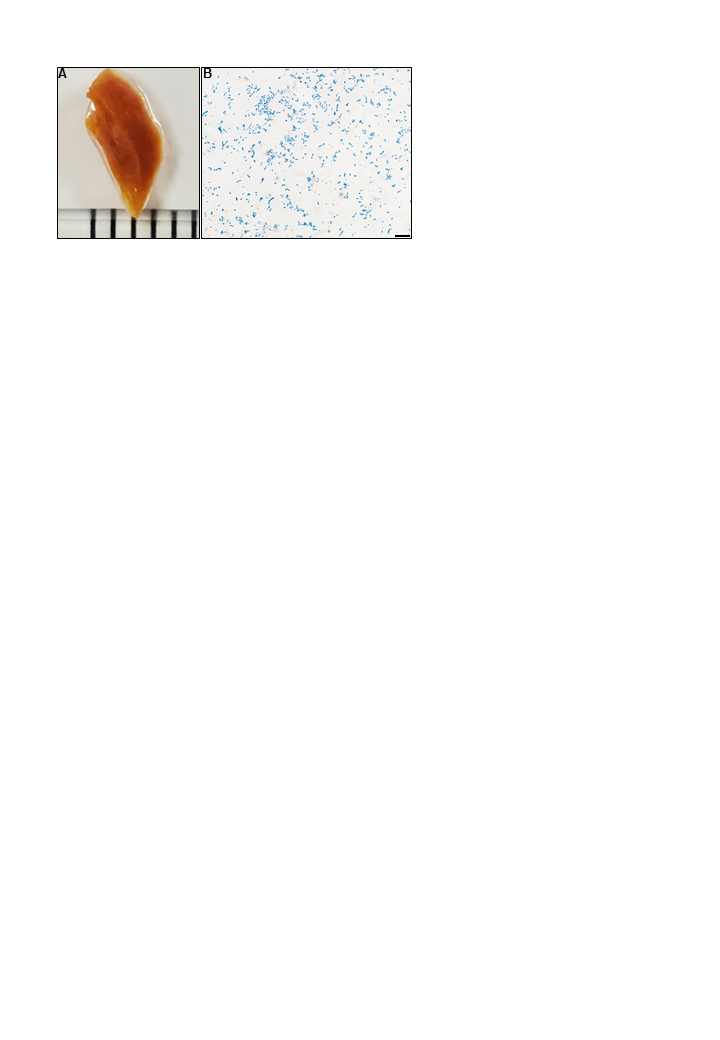

Supplement: Supplementary file 6 [file Image_1.TIF]
